# Understanding the USMLE journey of Brazilian medical students and graduates: a narrative review

**DOI:** 10.3389/fmed.2024.1484772

**Published:** 2024-10-17

**Authors:** Beatriz S. Garcia-Rosa, Lucas G. Urmenyi, Gabriel C. Santana, João Vitor M. Marques, Joao P. Miguez-Pinto, Clara Ramalho, Daniel Boczar, Bruno B. Andrade

**Affiliations:** ^1^Instituto de Pesquisa Clínica e Translacional, Medicina Zarns, Clariens Educação, Salvador, Brazil; ^2^Multinational Organization Network Sponsoring Translational and Epidemiological Research (MONSTER) Initiative, Salvador, Brazil; ^3^Curso de Medicina, Universidade Salvador, Anima Educação, Salvador, Brazil; ^4^Faculdade de Medicina, Universidade Federal do Rio de Janeiro, Rio de Janeiro, Brazil; ^5^Laboratório de Pesquisa Clínica e Translacional, Instituto Gonçalo Moniz, Fundação Oswaldo Cruz, Salvador, Brazil; ^6^Department of Surgery, Division of Plastic Surgery, Eastern Virginia Medical School, Norfolk, VA, United States

**Keywords:** medical school, USMLE, medical career, Brazil, medical residency education

## Abstract

Over the past decades, advances in medical technology and research have promoted the globalization of medicine, drawing medical students and physicians worldwide into seeking for better career and financial prospects, as well as enhanced clinical and research training abroad. The medical landscape in Brazil has been marked by significant challenges over the past years, particularly within the Unified Health System (SUS). Despite the expansion of medical schools and initiatives such as the *Mais Médicos* program arising as efforts to improve healthcare access and decrease regional inequalities in the country, such problems as under-resourced working environments, inadequate infrastructure, and unattractive financial compensation for both physician and research careers, have risen as persisting challenges. In the pursuit of improved conditions, the United States Medical Licensing Examination (USMLE) has emerged as an increasingly common pathway for those seeking to overcome these challenges. This review explores the motivations behind Brazilians pursuing the USMLE while examining structural and financial challenges within the country’s medical education and SUS landscape.

## Brazilian medical education

Admission to Brazilian medical schools relies on either qualifying exams termed “Vestibular” or the High School National Exam (ENEM). Vestibular exams are specific to each medical school and often created by private entities specialized in exam development, whereas the ENEM is a nationwide unified exam organized by the Brazilian government. Upon admission, the undergraduate course consists of a six-year curriculum (Table 1), with the last 2 years known as internships, involving clerkship rotations regulated by the National Curricular Guidelines for Medical Courses. The curriculum mandates that 70% of internship credit hours be dedicated to rotations in Internal Medicine, Surgery, Pediatrics, Gynecology and Obstetrics, Public Health, and Mental Health. More recent guidelines (2014) recommend a minimum of 30% of clerkship hours be spent in Family Medicine (primary care) and Urgent/Emergency care within the Brazilian Unified Health System (SUS) (1).

**Table 1:** Differences between the medical curriculum in Brazil and the United States.

Year of medical school curriculum	Brazil	United States of America
Year 1	Foundational curriculum	Foundational curriculum
Year 2	Foundational curriculum	Foundational curriculum / clinical clerkships
Year 3	Clinical knowledge	Clinical clerkships / post clerkship curriculum
Year 4	Clinical knowledge	Post clerkship curriculum
Year 5	Clinical clerkships	
Year 6	Clinical clerkships	

Upon completion, students receive their diplomas and are eligible to pursue residency programs. However, residency is not mandatory, and many begin work immediately after graduation, predominantly in primary care settings (2). In Brazil, obtaining a medical specialty can be achieved in various ways, each with specific criteria regulated by the Federal Council of Medicine (CFM) and the Brazilian Medical Association (AMB). The traditional route is through medical residency, an intensive practical training program lasting 2 to 5 years, depending on the specialty. This path is known for its structured training and rigorous supervision, providing comprehensive clinical experience.

Alternatively, a specialist title can be obtained through a certification exam, aimed at doctors with prior experience and practice in the field. Eligibility requires proof of internship completion or a minimum period of practice in the desired specialty, along with passing a theoretical and often practical exam organized by the corresponding specialty society.

Government financial aid programs such as the Student Financing Fund (FIES) and the University for All Program (PROUNI) support private medical education. FIES covers up to 100% of education costs for students who cannot afford them, with repayment over 18 years post-graduation at low interest rates. PROUNI offers full scholarships for students from low-income backgrounds. However, these programs benefit less than 20% of medical school seats (3), and many graduates leave medical school with significant debt, influencing their career choices and geographic distribution (4). Upon completion of a six-year medical program, the total debt incurred ranges from approximately US $172,800 to $316,800. This amount is often insufficient to be repaid with a residency salary of US $700 per month, particularly in larger cities where the cost of living is significantly higher.

In 2023, Brazil surpassed 380 medical schools, offering over 41,000 positions for medical students nationwide. Of these, only 23.2% (9,725 positions) are affiliated with government-run medical schools (3, 5). The establishment of new medical schools accelerated notably after the introduction of the Mais Médicos program in 2013 (6). At that time, Brazil had 1.8 physicians per thousand inhabitants, with only 8% practicing in small municipalities (6). Despite efforts by the program to reduce inequalities in access to medical education, most of the newly established schools were private institutions located predominantly in urban areas. By 2023, the physician ratio had risen to 2.41 per thousand inhabitants, but 53.5% of doctors remained concentrated in large cities (3), underscoring the ongoing regional disparities in distribution.

Furthermore, the uneven distribution of physicians and medical schools continues to create a workforce deficit that significantly affects the primary healthcare system (7, 8). For example, the shortage of physician’s forces municipalities to compete for a limited pool of professionals, leading to salary offers that often exceed municipal budgets. This unsustainable situation results in payment delays, contract breaches, and reduced working hours, all of which contribute to an inability to meet healthcare service demands and hinder access to quality care. As a result, numerous irregularities and legal challenges arise, with over half of the doctors leaving their positions within a year (5, 6). Consequently, access to quality healthcare in underserved regions remains severely compromised.

## Working conditions

Throughout the demanding journey of medical education, it is well established that the SUS serves as the principal training ground for universities, providing an essential environment for practical experience and student development (1). Notably, this exposure to the SUS often persists beyond graduation, as the system employs approximately 55% of the nation’s physicians (9). Thus, engagement with the public health system begins early in the educational process.

Medical students encounter a work environment characterized by significant challenges, including the need for improvisation due to shortages of supplies and inadequate maintenance of technical equipment (10, 11). This scenario necessitates navigating service deficiencies and making complex ethical decisions (12). Furthermore, insecurity within hospital units, combined with the demanding workload from multiple job commitments (with 50% of doctors working in 3 or 4 jobs) (13), leads to substantial psychosocial, physical, cognitive, and emotional strain. Consequently, this often results in professional burnout, diminished job satisfaction, and mental health issues, with approximately 80% of Brazilian doctors reporting professional fatigue and reduced income, and 24% of public sector physicians experiencing common mental disorders (13).

This challenging environment significantly influences medical students, leading many to reconsider their career trajectories due to the adversities they will face. The situation remains largely unchanged for physicians who have completed their residencies, prompting many newly graduated doctors to forgo residency programs altogether. This trend is evidenced by the decline in the number of first-year residents between 2019 and 2021 (5). Additionally, the financial burdens on graduates, coupled with the inadequate resident salary (approximately US $700 per month), further dissuade them from pursuing residency.

In the context of daily life in Brazil, this salary is generally insufficient. Monthly housing expenses range from US $300 to $600, while the cost of food typically falls between US $100 and $160 per month. Public transportation tickets are priced between US $0.90 and $1.20 each way, and owning a car entails a minimum cost of US $200 per month. In larger cities, housing alone can consume nearly the entire salary of a medical resident (14). Moreover, professionals with postgraduate degrees in clinical fields such as psychiatry, pediatrics, orthopedics, and internal medicine can attain the same positions designated for those with residency training, despite the latter’s more comprehensive and intensive curriculum.

Overall, early and sustained exposure to the SUS during medical training, along with the significant professional and financial challenges faced by healthcare workers, critically influences the career decisions of medical graduates in Brazil.

## Science

Historically, Brazil has maintained a low percentage of its GDP (Gross Domestic Product) allocated to research and development (R&D), typically around 1% (15). The private sector mirrors this trend with even lower investment levels. In recent years, federal funding for scientific endeavors has faced progressive reductions (16). These factors have significantly constrained universities and research institutions, leading to increasingly limited research opportunities. Consequently, students aspiring to pursue academic careers encounter barriers and challenges, often prompting them to seek opportunities abroad.

Given the inadequate compensation and absence of labor benefits, the notion of conducting scientific research as a Brazilian physician seems almost inconceivable. Postdoctoral scholarships, for instance, typically amount to approximately US $1,000 per month, necessitating full-time commitment, thereby rendering such roles unattractive due to the minimal professional returns relative to time invested (17).

Despite these challenges, individuals pursuing doctoral studies in Brazil confront yet another hurdle: the country’s research infrastructure, while featuring some cutting-edge centers in major cities and universities, generally suffers from limitations in terms of equipment, laboratories, and resources. This constrains the range of research projects available, discouraging students from exploring their preferred areas of interest.

Furthermore, Brazil’s healthcare system predominantly emphasizes preventive medicine, influencing medical school curricula and research funding priorities, thereby limiting the direction of different approaches.

## The brain drain phenomenon

The phenomenon of “fuga de cérebros,” or brain drain, has become a significant concern for Brazil, particularly in the medical field. This term refers to the emigration of highly skilled professionals from their home country to more developed nations in search of better opportunities and working conditions. Brazilian medical professionals are particularly drawn to practicing in the United States, driven by various factors previously discussed.

Although precise data on the number of doctors migrating abroad is lacking, there is a growing trend of individuals in the migration process and increasing interest among medical students in this issue. The situation becomes even more concerning when considering the emigration of researchers, contributing to what is known as the scientific diaspora.

Another factor driving the medical diaspora is the projected 15% increase in the total number of doctors in Brazil by 2030, without any current practical measures to improve working conditions (5). Additionally, various states in the U.S., such as Florida and Tennessee, have enacted laws allowing the acceptance of International Medical Graduates (IMGs) without the requirement of completing a new residency in the U.S. (18, 19). There has also been a 2.9% increase in the match rate of non-U.S. IMGs between 2020 and 2024, compared to a 12.3% increase in residency positions offered during the same period (20, 21).

The exodus of medical talent has profound implications for Brazil’s healthcare system. The loss of skilled doctors exacerbates the already strained public health infrastructure, reduced access to specialized care, and leads to an overall decline in the quality of healthcare services. Another significant issue is the lack of financial return from these doctors, many of whom complete their education through government scholarships and then choose to emigrate. This situation ultimately puts additional pressure on the remaining physicians and perpetuates a cycle of declining healthcare standards.

## The pathway and its barriers

Considering the aforementioned factors, it becomes evident that considerations such as scientific advancement and working conditions motivate both students and medical professionals to seek environments where these concerns are effectively addressed. Among available options, the American medical landscape stands out (22). However, this path to emigration presents challenges stemming from disparities in training, culture, language, and financial circumstances.

It’s notable that the USMLE imposes a standardized testing framework on its medical students, differing significantly from the norms typically seen in Brazilian universities (1). These variations reflect divergent medical education models between the two contexts. Brazil, grappling with complex social and economic challenges such as high-income inequality and prevalent diseases not seen in developed nations (23), focuses on preparing healthcare providers to meet the demands of its public health system (24). In contrast, American medical education where such social challenges are less pronounced, enables a focus on scientific advancement without the imperative of an assistance-oriented educational approach due to the absence of a public health system (25).

Moreover, the financial costs associated with validating a medical degree in the United States are substantial and often prohibitive for many Brazilian students. This investment encompasses registration fees for exams, travel and accommodation costs associated with testing centers, and expenses related to internships in American hospitals, all of which can be exacerbated by currency fluctuations.

Furthermore, the language barrier presents another obstacle; while only a small fraction of Brazilians are proficient in academic English (26), such proficiency is indispensable for validating a medical degree in the USA, thereby limiting the pool of eligible candidates. Moreover, in Brazil, there are no medical schools that offer their programs entirely or partially in English, all universities conduct their classes in Portuguese. This underscores the necessity for students who are interested in emigrating to independently acquire proficiency in medical English.

Analyzing the data from the NRMP 2024, it is evident that a significant proportion of IMGs apply for specialties that offer a higher number of available positions and lower competition. Specifically, 48.5% of the total IMG applicants in 2024 seek residency in internal medicine, followed by 11.4% in family medicine, 5.6% in pediatrics, and 4% in neurology (21).

Finally, despite all the differences discussed, the immediate financial return and the ability to practice clinically without the mandatory residency requirement are significant factors that make the Brazilian medical landscape attractive. This flexibility allows newly graduated doctors to start working immediately after completing medical school, providing quicker financial returns. Thus, while the path to validating a medical degree in the United States presents substantial challenges, the opportunities offered by the Brazilian medical system remain a viable option for many healthcare professionals.

## Conclusion

Despite their high level of education, many Brazilian doctors face limited opportunities for advanced training and specialization within the country, prompting them to seek further training abroad, particularly in the United States, which offers numerous residency programs, fellowships, and state-of-the-art facilities. The lack of opportunities, inadequate working conditions, and lower compensation in Brazil drive this trend. To address this, the Brazilian government must invest in its healthcare system, improve conditions for medical professionals, and create a more supportive environment for both practicing doctors and medical researchers.
